# Lasting Consequences of Traumatic Events on Behavioral and Skeletal Parameters in a Mouse Model for Post-Traumatic Stress Disorder (PTSD)

**DOI:** 10.1371/journal.pone.0042684

**Published:** 2012-08-22

**Authors:** Hongrun Yu, Heather Watt, Chandrasekhar Kesavan, Patrick J. Johnson, Jon E. Wergedal, Subburaman Mohan

**Affiliations:** 1 Musculoskeletal Disease Center, Jerry L. Pettis Memorial VA Medical Center, Loma Linda, California, United States of America; 2 Department of Medicine, Loma Linda University, Loma Linda, California, United States of America; Wayne State University, United States of America

## Abstract

**Background:**

Post-traumatic stress disorder (PTSD) is an anxiety disorder that not only affects mental health, but may also affect bone health. However, there have been no studies to examine the direct relationship between PTSD and bone.

**Methodology/Principal Findings:**

We employed electric shocks in mice to simulate traumatic events that cause PTSD. We also injected the anxiogenic drug FG-7142 prior to electric shocks. Electric shocks created lasting conditioned fear memory in all mice. In young mice, electric shocks elicited not only behavioral response but also skeletal response, and injection of FG-7142 appeared to increase both types of response. For example in behavioral response within the first week, mice shocked alone froze an average of 6.2 sec in 10 sec tests, and mice injected with FG-7142 froze 7.6 sec, both significantly different (*P*<0.05) from control mice, which only froze 1.3 sec. In skeletal response at week 2, shocks alone reduced 6% bone mineral content (BMC) in total body (*P* = 0.06), while shocks with FG-7142 injection reduced not only 11% BMC (*P*<0.05) but also 6% bone mineral density (BMD) (*P*<0.05). In addition, FG-7142 injection also caused significant reductions of BMC in specific bones such as femur, lumbar vertebra, and tibia at week 3. Strong negative correlations (R^2^ = −0.56, *P*<0.05) and regression (y = 0.2527−0.0037 * x, *P*<0.01) between freezing behavior and total body BMC in young mice indicated that increased contextual PTSD-like behavior was associated with reduced bone mass acquisition.

**Conclusions/Significance:**

This is the first study to document evidence that traumatic events induce lasting consequences on both behavior and skeletal growth, and electric shocks coupled with injection of anxiogenic FG-7142 in young mice can be used as a model to study the effect of PTSD-like symptoms on bone development.

## Introduction

Post-traumatic stress disorder (PTSD) is an acquired anxiety disorder developed after experiencing a traumatic event that involves the threat of death or injury. It is characterized by frequent re-experiencing of the trauma with intense fear and helplessness, persistent avoidance of associated stimuli, and hyperarousal [1], [2]. PTSD can be classified on the basis of the traumatic events into those occurring after a single traumatic event and those as the result of a series of negative events that build up to the traumatic threshold. PTSD can also be grouped by the time of occurrence of the symptoms into the following categories: acute, chronic, delayed, and both delayed and chronic. In acute PTSD patients, the symptoms appear shortly after the trauma, and do not last longer than a certain period of time, a few months for example. Chronic PTSD sufferers display symptoms beyond a certain period of time of the original trauma. Another characteristic of PTSD is that onset of the condition can also be delayed for an unpredictable period of time. In the United States, an estimated 70% of the adults have experienced at least one traumatic event in their lifetime, and 20% of these people will go on to develop PTSD [3]. Other surveys of PTSD prevalence found that 5–6% of men and 10–14% of women have had PTSD at some time during their lifetime [4], [5], [6]. PTSD significantly impacts a person's normal life and reduces its quality. Elucidation of biological changes related to this disease and mechanisms that contribute to these changes is important for identifying effective strategies to combat PTSD symptoms.

There is indirect evidence that PTSD may also affect bone health. First, individuals who develop PTSD have an increased risk of major depression [7]. Patients with depression have a 6–15% lower bone mineral density (BMD) and an increased risk of osteoporotic fractures [8], [9]. Second, serum levels of stress hormones, such as glucocorticoids, are known to be altered in patients with possible PTSD [10], [11], [12], [13]. Glucocorticoid-induced osteoporosis is one of the common forms of secondary types of osteoporosis [14]. Third, animal studies link chronic stress derived depression, a related disorder of PTSD, to decreased bone formation. Mice subjected to chronic mild stress, an established bone model of depression, exhibit impaired bone mass and structure caused by decreased bone formation [9]. Fourth, clinical studies associate possible trauma exposure or PTSD history to decreased bone formation. For example, iliac crest bone biopsies from 17 Gulf War veterans showed a decreased bone formation rate and a decreased trabecular bone volume compared to age-matched controls [15], [16]. An evaluation of 241 Vietnam-era male repatriated prisoners of war with and without the lifetime diagnosis of PTSD revealed that the repatriates with a lifetime history of PTSD had a significantly lower BMD at the hip compared to both repatriates without a life long history of PTSD and control group of normal subjects [17].

Although emotional disorders such as depression and anxiety are known to be associated with reduced bone density in clinical studies [18], [19], they are quite different disorders from PTSD. For example, depression is not necessarily part of PTSD symptoms, and an anxiety may not have an origin in a traumatic event. As such, there are no studies to examine the direct relationship between PTSD and bone. The goal of the current study was to determine both behavioral response and skeletal response to traumatic events that cause PTSD in mice, and to develop a suitable model that can be used to measure the effect of PTSD on bone metabolism.

Previous animal studies of PTSD mainly employed rodents. Common stressors to simulate traumatic events include electric shocks delivered to the animal's feet from cage floor [1], [2], [3], [20], [21], [22], exposure to feline predators such as cats [23], [24], physical restraint [25], [26], and immobility [27]. We chose electric foot shocks in mice as the stressor. We tested electric shocks alone and electric shocks coupled with the prior injection of the anxiogenic drug FG-7142, which has been shown to induce anxiety-related behavioral response [28]. We have found that electric shocks alone produced sustained contextual fear expressed in behavioral response when applied to both pre-pubertal and adult mice. However, a skeletal response was seen in mice exposed to electric shocks only before but not after pubertal growth spurt. Furthermore, FG-7142 injection appeared to increase the effect of electric shocks in both behavioral and skeletal responses. We believe that electric shocks coupled with prior injection of anxiogenic drugs in mice can be a useful model to determine the efficacy of therapeutic approaches in mitigating PTSD related mental and bone diseases.

## Methods

### Animals used

All experiments were carried out with C57BL/6J mice, purchased from the Jackson Laboratory (Bar Harbor, Maine). We used only female mice since they could be group housed. Animals arrived at least one week before the experiment, and were allowed to acclimate to local environment. For young mice used at three weeks of age, pups were shipped with the mothers at two weeks of age, and weaned onsite at three weeks. All animals were housed 3 per cage at the Animal Research Facility of Jerry L. Pettis Memorial VA Medical Center under the standard condition of 14 h light, 10 h darkness, ambient temperature of 20°C, and relative humidity of 30–60%. All experimental protocols were in compliance with the established animal welfare regulations, and approved by the animal research committee of Jerry L. Pettis Memorial VA Medical Center.

### Experiments

**Figure 1:**
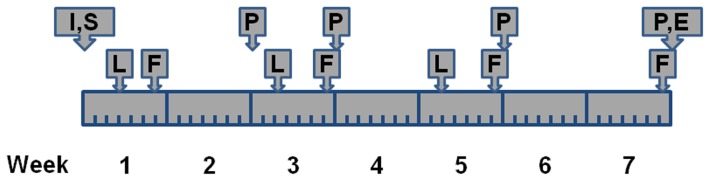
Illustration of schedule of experiment B. I = intraperitoneal injection of FG-7142; S = electric shocks; L = light/dark test; F = freeze test; P = PIXImus scan; E = Euthanasia. The light/dark test was conducted on day 3, and repeated every other week twice; the freeze test on day 6, and repeated every other week for three times; the PIMImus scan at week 2 and 3, and repeated every other week for two more times.

Two experiments were carried out. In experiment A, we used adult mice, and measured bone mass parameters six weeks after electric shocks. No significant effect of electric shocks was observed on bone phenotypes. Therefore, we changed to younger mice in experiment B, and used longer electric shocks, and started measuring bone mass parameters early at week 2 (**Table 1**). In addition, anxiogenic drug FG-7142 was also injected prior to electric shocks for some mice in experiment B. Behavioral tests were conducted every other week for both experiments, and bone mass parameters were measured during the same period. The schedule of activities and test events for experiment B is shown in **Figure 1**. The detailed methods of shock treatment, drug injection and conducted tests and measurements are described below. All mice in each experiment were handled in the exactly same manner. The equipment was thoroughly cleaned during each test with 70% ethanol before each new animal was introduced.

**Table 1:** Information regarding mice used, shock treatment, and phenotypes determined for each experiment.

Exp	Animal	Sex	No. of animals		Electric shocks		Weeks when phenotypes determined		
	age								
			Shocked	Control	Duration	Times	Light/dark test	Freeze test	Bone mass
	(weeks)		(injected vs. not)		(sec.)				
A	9	F	24	12	2	3	1, 3	1, 3, 5	6
B	3	F	24 (12 vs. 12)	12	3	3	1, 3, 5	1, 3, 5, 7	2, 3, 5, 7

### Electric shocks

We used one-time electric foot shocks as the stressor to mimic a single traumatic event. The shocks were conducted in a Freeze Monitor (San Diego Instruments, San Diego, CA). The inside dimension of the Freeze Monitor chamber is 10″×10″×7 1/2″. The chamber has transparent walls and a metal rod floor. The metal rod floor consists of 1/8″ thick grids spaced at 5/16″. The Freeze Monitor is programmable through a computer to deliver stimuli such as shocks and sound. Mice were shocked one at a time. We carried out three shocks spaced 1 min apart with intensity of 1.5 mA and duration of 2 or 3 sec (**Table 1**). Each shock session lasted for 6 min. First, mice were allowed to explore the chamber for 3 min. Then, the shocks were carried out. Each shock minute was started with a cue of an acoustic tone at 85 dB for 20 sec. The actual shock was delivered at the end of and co-terminated with the cue presentation. Control animals went through the same procedure without the shocks.

### Drug injection

We used the anxiogenic drug, N-methyl-beta-carboline-3-carboxamide (FG-7142) (Sigma Aldrich, St. Louis, MO) in half of shocked mice (N = 12) in experiment B. First, the dissolvent Tween 80 (Sigma Aldrich, St. Louis, MO) was diluted to 0.1% in phosphate buffered saline. FG-7142 was then dissolved in the 0.1% Tween 80 as described previously [29], [30]. We used a dose of 40 mg/kg based on body weight [31], [32]. The drug was injected intraperitoneally within 36–60 min prior to electric shocks. The injection volume was 10 ml/kg [30]. The shocked alone and control mice were injected with the vehicle (0.1% Tween 80).

### Light/dark test

The light/dark box test measures the exploratory behavior of rodents in novel and unprotected environments. It is based on their nocturnal nature and their innate aversion to brightly illuminated areas. Reduced exploration can be considered as part of the PTSD-like symptoms related to the sensitized fear. Mice with greater fear and anxiety will presumably spend less time in the light. The light/dark test was carried out in a box similarly as previously described [33]. The box consists of two connecting chambers with one dark and one brightly lit. We made the box from wood. The inside dimension of both chambers measures 7.5″ long, 7.5″ wide and 5.5″ high. There is a small rectangular opening 2.5″ wide and 3.0″ high that connects the two chambers. The cover top for the light chamber is made from transparent plastic with ventilation holes. A 100 W light bulb 16″ above the plastic cover provides illumination at intensity of 700 lux on the floor of the light chamber. Each test session lasted for 5 min. The animal was first placed in the light chamber. The animal's movement into the dark chamber and back into the bright chamber was recorded by the video-recorder installed above the box. The video-tape was reviewed and scored later. The accumulated time (sec) a mouse spent in the light chamber was determined.

### Freeze test

Freezing is a behavior defined as the absence of any visible movement except that required for respiration, and is a natural response to fear. Freezing behavior has been used previously and proved to be a useful measure in a number of animal-based PTSD studies [34], [35], [36], [37]. The freeze test, which was used to determine the conditioned fear memory, was conducted in the Freeze Monitor - the same equipment used to deliver the initial electric shocks. Animal movement inside the Freeze Monitor chamber is detected by the infra-red photo-beams in a 16×16 array with the beam spacing of 1/2″ in each direction. The 6 min test session was the same as the shock session in terms of the schedule of cue presentation except that there were no electric shocks delivered. First, a mouse was allowed to explore the chamber for 3 min. Then, each of the 3 test min was divided into 6 10-sec periods for recording the latency of animal movement, which is considered the freezing time. If the latency is 0, i.e. the animal did not move at all, the freezing time is the maximum, i.e. 10 sec. Since only the second period was associated with both cue presentation and the initial shock, the freezing time of this period averaged over the 3 test min was used as a measure of the freezing behavior.

### Bone mass measurements

Bone mass parameters were measured using a PIXImus densitometer (LUNAR Corp., Madison, WI) as described previously [38]. The procedure was carried out under anesthesia. To induce anesthesia, a mouse was first placed in a Plexiglass container for 2–3 min. The container was connected to an anesthesia machine that provided constant flow of isoflurane gas (2–4% supplied in 1 liter/min oxygen). Then, the anesthetized mouse was transferred to a special tray, put in the imaging area of the PIXImus desitometer, and hooked to a mask for anesthesia maintenance. The mask was also connected to the anesthesia machine that supplied 3% isoflurane gas with 0.5 liter/min oxygen. X-ray scanning was carried out by the PIXImus desitometer. The image was analyzed on a connected computer. To obtain bone mass parameters of total body, a region of interest (ROI) rectangle was moved and re-sized to cover the whole body, and the animal's head was set as an exlusion zone. Bone mass parameters of specific bones such as femur, lumbar vetebra and tibia were obtained by similarly moving the ROI rectangle to the specific region. The PIXImus software automatically calculated and recorded the data in an Excel file. We used three bone mass paramters: bone mineral content (BMC), bone mineral density (BMD) and total body area (T Area).

### Data analyses

Data analyses proceeded in two steps. First, general analyses were carried out to assess the effect of overall shock stress, where both experiments were included, and all shocked mice in experiment B were grouped together as one treatment regardless of FG-7142 injection. Then, specific analyses were carried out for experiment B to assess the effect of FG-7142, where shocks alone and shocks coupled with FG-7142 injection were treated as two separate treatments, and to determine the relationship between behavorial response and skeletal response. Although no significant difference by chance between shocked and control mice in body weight was found during analyses, we did find such a significant difference within shocked mice between those shocked alone and those injected with FG-7142. This body weight difference began with ordered pups due to varying litter sizes, and decreased as the experiment progressed. Because BMC from PIXImus is known to be sensitive to body weight, all BMC analyses comparing shocks alone and shocks with FG-7142 injection were based on body weight adjusted values. For this purpose, we used a modified version of a previous regression formulae [39]: adjusted value = un-adjusted value/(predicted value×un-adjusted value mean), where predicted value = (intercept+slope×body weight). All data analyses were performed using the Statistica software (StatSoft, Inc., Tulsa, OK). Variance analyses included two-way ANOVA (shock treatments x time points) for paramters with values at multi-time points, and one-way ANOVA for paramters at a single time point. Significances (*P* values) for all comparisons between a treatment and the control or between two treatments were obtained from a post hoc Fisher LSD test. Correlation analysis was carried out by calculating Pearson product-moment correlation coeffciensts (R^2^).

## Results

### Effect of overall electric shock stress

In the light/dark test, there were no significant differences between the shocked and control mice in the accumulated time they stayed in the bright chamber at any time point for both experiments (**Table 2**). However, when data from both experiments were combined, the shocked mice spent significantly less time in the bright than the control mice within the first week (92.9 vs. 111.5 sec, *P* = 0.05). These results suggest that with a reasonably large number of animals, the shock treatment can produce significant fear and anxiety in mice that can be measured by this test within the first week.

**Table 2:** Accumulated time (sec) spent in the bright chamber out of a 5 min test period in the light/dark test.

Exp	Week 1			Week 3			Week 5		
	Shocked	Control	*P*	Shocked	Control	*P*	Shocked	Control	*P*
A	88.0±5.5	105.3±6.4	0.15	92.6±8.1	80.5±10.7	0.30			
B	98.0±7.5	117.8±10.4	0.17	119.0±10.1	109.3±15.0	0.51	95.2±7.4	109.9±10.9	0.32
Combined	92.9±4.7	111.5±6.1	0.05	104.9±6.6	94.9±9.5	0.30	95.2±7.4	109.9±10.9	0.29

Values represent mean ± SEM.

The “Shocked” group also includes mice injected with FG-7142.

In the freeze test, there were significant differences between the shocked and control mice in the freezing time (*P*<0.01) at all time points for both experiments (**Table 3**). The shocked mice froze a longer period than the control mice, and the effect lasted for seven weeks. However, the freezing time for the shock mice appeared to decrease over time, while there was not much change for the control. For example in experiment A, where adult mice were used, the freezing time ratio between the shocked and control mice was 3.28:1 and 2.65:1 for week 1 and 3, respectively, while in experiment B using young mice, the ratio was much higher at 5.33:1 and 3.67:1. Two-way ANOVA showed that the main effect of shock treatment was significant in both experiment A (F[1,102] = 46.3, *P*<0.01) and experiment B (F[1,127] = 97.75, *P*<0.01). The main effect of time points was also significant in experiment B (F[3,127] = 7.04, *P*<0.01). These results mean that shock treatment caused significant increases in mouse freezing behavior. The increased freezing behavior, however, was diminished in a time-dependent manner, especially in young mice.

**Table 3:** Average time of freezing (sec) out of 10 sec test periods in the freeze test.

Exp	Week 1		Week 3		Week 5		Week 7	
	Shocked	Control	Shocked	Control	Shocked	Control	Shocked	Control
A	3.84±0.37**	1.17±0.13	4.13±0.35**	1.56±0.40	4.71±0.47**	1.95±0.31		
B	6.88±0.41**	1.29±0.23	4.74±0.46**	1.29±0.23	3.66±0.33**	1.65±0.40	2.89±0.57**	1.24±0.17

Values represent mean ± SEM.

** indicates significance at *P*<0.01 from the control.

The “Shocked” group also includes mice injected with FG-7142.

**Figure 2:**
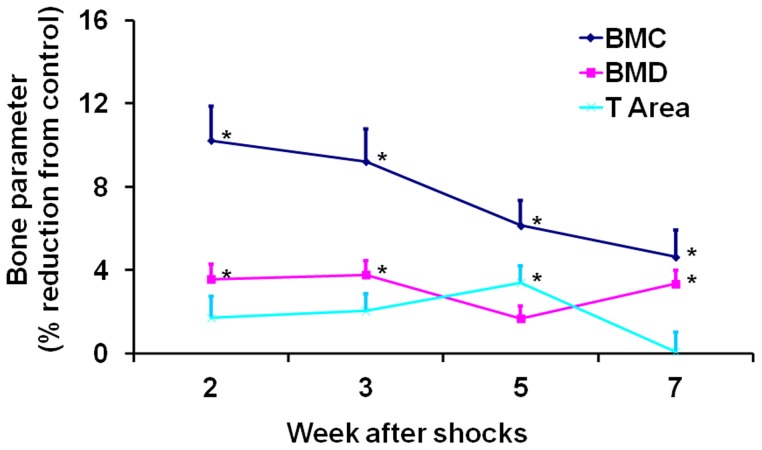
Reductions of bone mass parameters in total body in shocked mice. The shocked mice also include those injected with FG-7142. For each parameter, values are means and S.E.M of percentage reductions compared to the control. An asterisk on the right indicates a significant difference from the control (*P*<0.05).

We did not find any significant differences between the shocked and control mice in bone mass parameters as measured 6 weeks after electric shocks in experiment A (data not shown). However, we did observe significant differences in experiment B (**Figure 2**). In total body, BMC was affected most, followed by BMD, while T Area was least affected. BMC of the shocked mice was reduced 10% (*P*<0.05) and 9% (*P*<0.05) compared to the control mice at week 2 and 3, respectively. BMD was reduced about 4% at both time points (both *P*<0.05). Like the freezing behavior, these reductions were at the maximum at week 2, and appeared to decrease over time, especially for BMC. Two-way ANOVA showed that the main effect of shock treatment was significant for all three parameters (BMC: F[1,126] = 33.49, *P*<0.01; BMD: F[1,126] = 28.90, *P*<0.01; T Area: F[1,126] = 6.32, *P* = 0.01). One-way ANOVA using the percentage values presented in Figure 2 showed that the effect of time points was close to significance for BMC (F[3,82] = 2.42, *P* = 0.07). These results indicate that electric shocks had significant effect on bone mass acquisition in young mice that started as early as week 2 after electric shocks. Reduction of BMC acquisition appeared to diminish over time as the animals were recovering from electric shocks.

### Electric shocks alone vs. electric shocks coupled with injection of FG-7142

**Figure 3:**
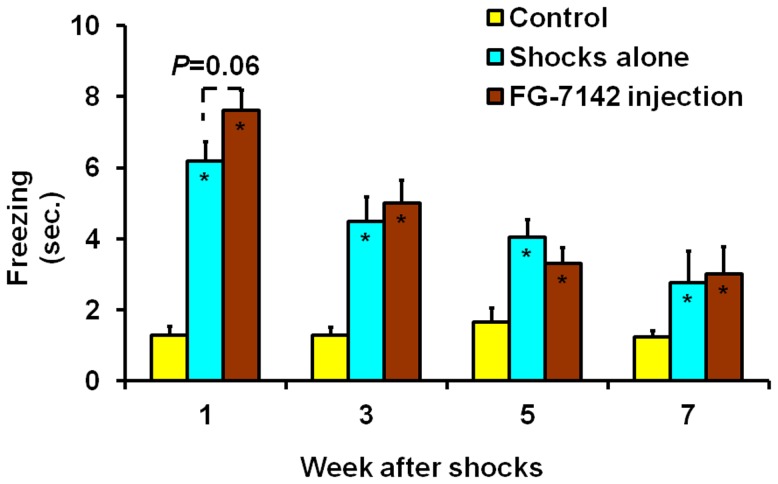
Effect of electric shocks alone and electric shocks plus FG-7142 injection on freezing behavior. Values are means and S.E.M. of freezing duration (sec) during 10-sec test periods. The significance of each treatment compared to the control at each time point is indicated by an asterisk for *P*<0.05 inside the corresponding bar. The significance between two treatments is indicated by the actual *P* value above the corresponding bars.

In experiment B, both treatments, shocks alone and shocks coupled with injection of FG-7142, showed significant effect on the freezing behavior at all time points (**Figure 3**). For example, within the first week, mice treated with shocks alone froze an average of 6.2 sec out of 10 sec tests, mice treated with both electric shocks and FG-7142 injection froze 7.6 sec, while control mice only froze 1.3 sec. Both treatments were significantly different (both *P*<0.05) from the control, while the FG-7142 injected treatment coupled with shocks was also close to statistical significance (*P* = 0.06) compared to shocks alone. These results show that electric shocks alone had significant effect on mouse freezing behavior, and there was a trend that prior injection of FG-7142 increased the effect of electric shocks on this behavior within the first week.

**Figure 4:**
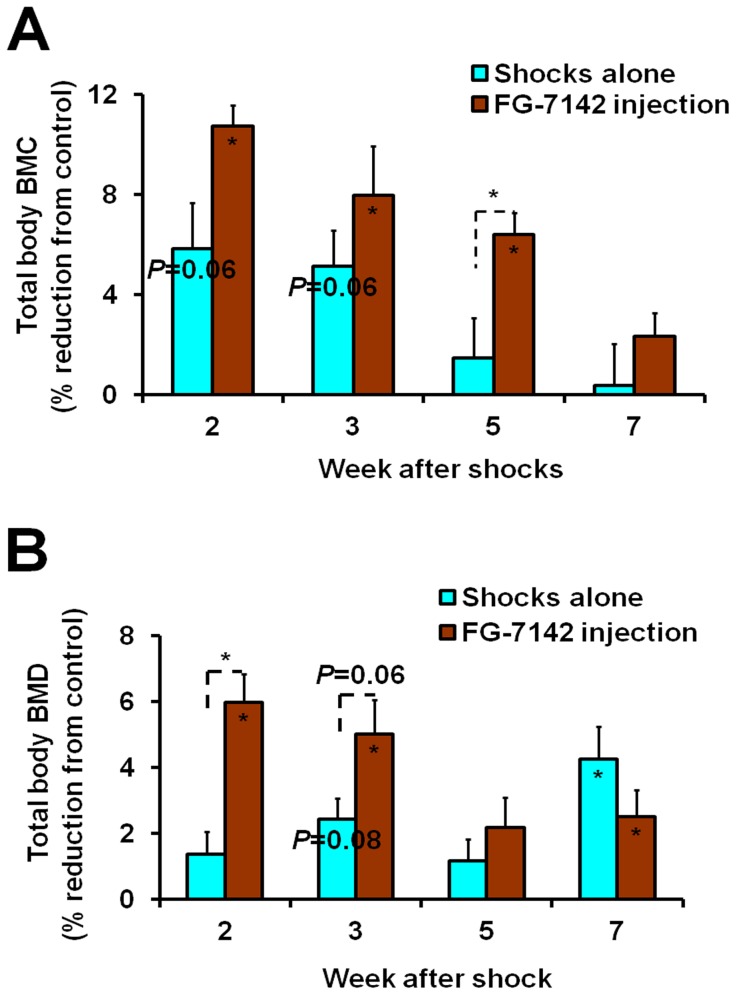
Reductions of bone mass parameters in total body caused by electric shocks alone and electric shocks plus FG-7142 injection. (**A**) BMC. The percentage reductions for BMC were calculated from body weight adjusted values. (**B**) BMD. Values for each parameter are means and S.E.M of percentage reductions compared to the control. The significance of each treatment compared to the control at each time point is indicated by the actual *P* value or an asterisk for *P*<0.05 inside the corresponding bar. The significance between two treatments is indicated by the actual *P* value or an asterisk for *P*<0.05 above the corresponding bars.

**Figure 5:**
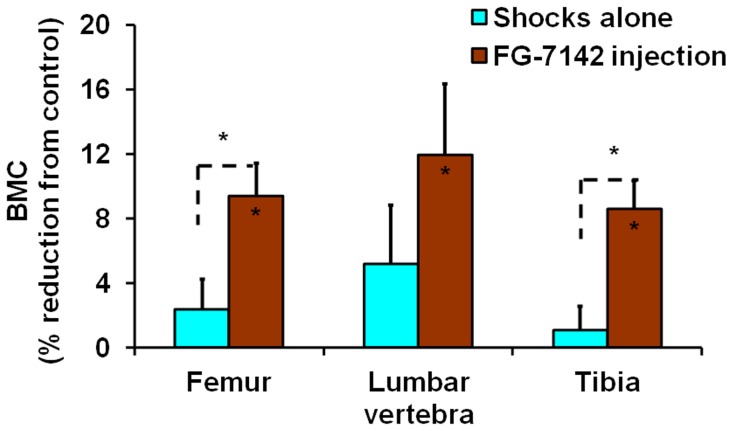
Reductions of BMC in specific bone sites at week 3 caused by electric shocks alone and electric shocks plus FG-7142 injection. **Values are means and S.E.M of percentage reductions compared to the control.** The percentage reductions were calculated from body weight adjusted BMC values. The significance of each treatment compared to the control for each parameter is indicated by an asterisk for *P*<0.05 inside the corresponding bar. The significance between two treatments is indicated by an asterisk for *P*<0.05 above the corresponding bars.

Both treatments also resulted in decreased bone mass acquisition, but there was stronger effect for shocks coupled with FG-7142 injection. For example, while shocks alone caused 5.8% and 5.1% reductions (both *P* = 0.06) in total body BMC at week 2 and 3, respectively, shocks with FG-7142 injection caused 10.8% and 8.0% reductions (both *P*<0.05) in the same periods (**Figure 4A**). The difference between the two treatments was significant (*P*<0.05) at week 5. In total body BMD, while there was only 2.4% reduction (*P* = 0.08) for shocks alone at week 3, shocks coupled with FG-7142 injection caused 6.0% and 5.0% reductions (*P*<0.05 and  = 0.06, respectively) for week 2 and 3 (**Figure 4B**). The differences between the two treatments were significant (*P*<0.05) at week 2 and close to significance (*P* = 0.06) at week 3. In addition, in specific bones, although there were no significant reductions in BMC for shocks alone at week 2 and 3, shocks coupled with FG-7142 injection caused significant BMC reductions at week 3 (9.4% for femur, 12% for lumbar vertebra and 8.6% for tibia, all *P*<0.05) (**Figure 5**). The differences between the two treatments were significant in femur and tibia (both *P*<0.05). To summarize, these results indicate that electric shocks alone reduced bone mass acquisition in young mice, and FG-7142 injection increased this effect by not only reducing total body BMC and BMD to a greater degree but also reducing BMC of specific bones.

### Relationship between behavioral and skeletal responses

**Figure 6:**
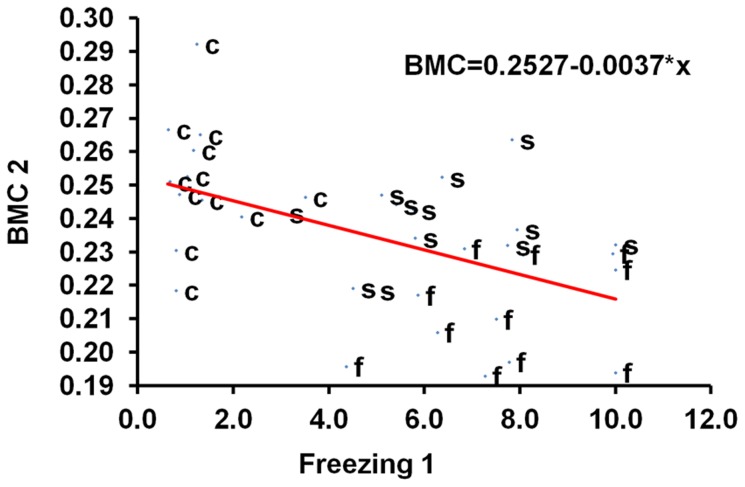
Regression using total body BMC as the dependent variable and freezing time as the independent variable. “BMC 2” refers to the total body BMC (g) at week 2, and “Freezing 1” the freezing time (sec) at week 1. s = electric shocks alone, f = electric shocks plus FG-7142 injection, c = control. R^2^ = 0.261, N = 35, *P*<0.01.

The relationship between behavioral measures and bone mass parameters was determined by a correlation analysis using the significant behavioral measure, the freezing time, and the most sensitive bone mass parameter, the total body BMC, as shown in **Table 3** and **Figure 2** **, respectively**. Most values of the freezing time at different time points were significantly correlated with each other. For example, the correlation coefficients (R^2^) for the freezing time at week 1 and 3 (“Freezing 1” and “Freezing 3”) was 0.56 (*P*<0.05) (**Table 4**). Similarly, values of total body BMC were also significantly correlated across different time points. The highest correlation coefficient was 0.76 (*P*<0.05) between BMC at week 2 and 5 (“BMC 2” and “BMC 5”, respectively). More importantly, significant negative correlations were observed between the freezing time and total body BMC. For example, the correlation coefficients for “Freezing 1” and “Freezing 3” with “BMC 2” were −0.52 and −0.55 (all *P*<0.05), respectively. The relationship between behavioral response and skeletal response to shock treatment was further carried out by a regression analysis using “BMC 2” as the dependent variable (y) and “Freezing 1” as the independent variable (x). We obtained a regression: y = 0.2527−0.0037 * x (**Figure 6**). Both the constant (0.2527) and coefficient (−0.0037) were statistically significant (*P*<0.01). Taken together, the significant negative correlations and regression between the freezing time and total body BMC suggest that there is a negative relationship between the contextual PTSD-like behavior, which we believe the freezing time measures, and bone mass acquisition, and reduced bone mass acquisition can be predicted in part from increased contextual PTSD-like behavior.

**Table 4:** Correlation coefficients (R^2^) between freezing time and total body BMC based on correlation analysis.

	BMC 2	BMC 3	BMC 5	BMC 7	Freezing 1	Freezing 3	Freezing 5
BMC 3	**0.592**						
BMC 5	**0.760**	**0.490**					
BMC 7	**0.559**	**0.627**	**0.711**				
Freezing 1	**−0.519**	−0.334	**−0.517**	**−0.350**			
Freezing 3	**−0.554**	**−0.362**	**−0.383**	**−0.394**	**0.561**		
Freezing 5	−0.232	−0.280	0.006	0.148	**0.345**	**0.375**	
Freezing 7	−0.242	−0.248	**−0.441**	−0.299	0.328	0.091	0.135

Numbers following the “Freezing” or “BMC” parameter indicate the week when the data were collected. For example, “Freezing 1” is the freezing time for week 1 after shock treatment.

**Bold** font indicates significance at *P*<0.05.

**Figure 7:**
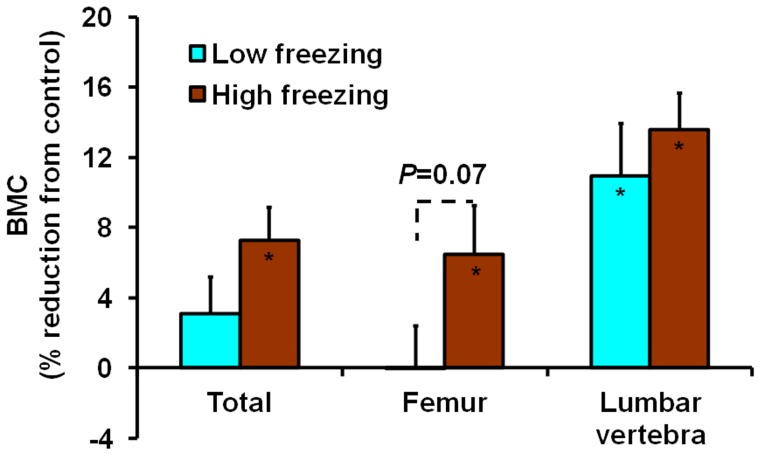
Comparisons of BMC reductions in total body and specific bone sites between low and high freezing response groups of mice. All parameters were obtained at week 7. Values are means and S.E.M of percentage reductions compared to the control. The significance of each group compared to the control for each parameter is indicated by the actual *P* value or an asterisk for *P*<0.05 inside the corresponding bar. The low and high freezing response groups were defined as having the freezing time in the lowest and the highest 33% percentiles, respectively. The mean freezing time in the low response, high response and control group was 1.15, 5.35 and 1.24 sec, respectively. While there was a significant difference between the high response and control group for the freezing time, the difference between the low response and control group was not significant.

We also examined the relationship by dividing the shocked mice into three groups based on the freezing time at completion of the experiment (i.e. week 7): low (low 33% percentile), medium, and high (top 33% percentile) freezing responses. Even though the correlation between “Freezing 7” and “BMC 7” was weak with a non-significant correlation coefficient of −0.30 (**Table 4**), the high response group still had significant BMC reductions in total body and femur (7.3%, *P*<0.05; 6.5%, *P*<0.05, respectively) compared to control mice, while there were no significant reductions in the low response group (**Figure 7**). The difference between the low and high response groups in femur was also close to significance (*P* = 0.07). In lumbar vertebra, the reduction of BMC in the high response group was higher (13.6%) than the low response group (11.0%), although both numbers were significant (both *P*<0.05). These data show that the high freezing response mice had increased BMC reductions compared to the low response mice.

## Discussion

We chose electric foot shocks as the stressor since other models involve prolongation and repetition of exposure in order to vary the stressor intensity, which induce an onset of habituation, and prevent or even invert a dose-response effect [40]. The intensity of electric shocks can be conveniently varied by changing the current, duration and number of shocks. Even though multiple electric shocks are used, they are usually delivered in a short time, and can be considered to mimic a single traumatic event. Therefore, electric shocks in rodents have been widely used to model PTSD [40], [41], [42]. Many different methods of electric shocks have been used. For example in one study, the C57BL/6 mice were shocked at 0.5 mA for 7.5-sec every 2 min with the whole procedure lasting up to 12 hours [43]. In another study using the C3H/HeN mice, a foot shock pulse with duration of 0.5 sec was given every 5 sec for 30 min per day for five consecutive days [44]. Recent studies, however, use a fewer number of shocks (1–2), stronger current (1.5–3 mA) and shorter duration (2–10 sec) [41], [45], [46], [47]. Accordingly, we used 3 shocks (1.5 mA, 2–3 sec) spaced 1 min apart. We tried to measure the sensitized (non-associative) fear memory generated by this shock regimen using the light/dark test, the elevated plus maze test, and the startle test. In the light/dark test, there only appeared to be effect within the first week. In the other tests, the effect was either only significant within the first week like the light/dark test or inconsistent between different time points (data not shown). These results could be due to two factors. First, the intensity of electric shocks might not be strong enough to produce detectable lasting sensitized fear. Indeed, a much stronger shock regimen at 3 mA for 10 sec has been recently employed on adult mice [41]. Second, the sensitivity of behavioral tests was not strong enough to detect the sensitized fear memory. These tests suffered from the weakness of animal habituation due to repeated measurements. However, our shock regimen did produce significant effect on the freezing behavior, which is a measure of the conditioned (associative) fear memory. The symptoms of PTSD are divided into those related to the memory of the trauma and those generalized and un-related directly to the trauma memory [40]. This study suggests that electric shocks at commonly used intensities to model PTSD may be particularly applicable to the trauma related fear memory.

In order to identify an optimal traumatic stress regimen that can produce detectable lasting fear memory, preferably sensitized fear memory, that characterizes PTSD, we explored ways of using pharmacological stressors to chemically treat the animals to create anxiety. FG-7142 is such a drug that acts as a partial inverse agonist of benzodiazepine receptors [48]. The benzodiazepine receptors are allosteric modulatory sites on GABAα receptors, which, upon activation, selectively conduct Cl- through their pores, resulting in hyperpolarization of neurons, which in turn causes an inhibitory effect on neurotransmission. As a partial inverse agonist, FG-7142 mitigates this inhibition, and evokes behavior with increased anxiety and hyper-vigilance. Thus, FG-7142 is an anxiogenic agent that may be suitable for use in conjunction with electric shocks. Previous studies found that it induced anxiety-related behavioral response [28], and significantly reduced social interaction in mice [49]. The drug also caused severe anxiety in humans [50]. We injected it intraperitoneally at a dose of 40 mg/kg 36–60 min before electric shocks. The freezing behavior of FG-7142 injected mice was increased only modestly and immediately compared to that of mice shocked alone. However, reductions of BMC and BMD in total body as well as in specific bones such as femur and tibia were significantly greater and lasted longer. Thus, the injection of FG-7142 appeared to enhance the traumatic stress regimen by increasing the conditioned fear memory in behavioral response and by reducing bone mass acquisition in skeletal response. Our assumption regarding FG-7142 was that pre-treatment with the drug would create temporary anxiety that enhances the effect of electric shocks, and it itself would not have long lasting effect on either animal behavior or bone phenotypes. Our rationale for this assumption is as follows. First, FG-7142 alone could not, in any way, affect the freeze test, the only behavioral test that showed consistent significant differences, since a response to this test was prompted by audio cues associated with electric shocks. Second, literature search did not produce any studies attributing effect on bone to FG-7142. Third, we only injected a single dose. Effect of FG-7142 on behavior or otherwise, if there was any, was likely transient. However, the study suffered a weakness by lacking a treatment of FG-7142 injection alone to prove the assumption.

Bone mass parameters in experiment A, where older mice were used, did not show significant changes at week 6 after electric shocks, the only time point when these parameters were collected. However, the results of experiment B using younger mice indicated that three electric shocks (1.5 mA, 3 sec, 1 min apart) significantly reduced BMC and BMD in total body 2–3 weeks after the treatment. The injection of FG-7142 prior to electric shocks enhanced this effect with increased reductions of BMC and BMD in total body. In addition, significant reductions of BMC in femur, lumbar vertebra and tibia were also observed in FG-7142 injected mice at week 3. To the best of our knowledge, this study is the first animal study to document evidence of the effect of traumatic events on bone mass acquisition. It is known that bone mass continues to accrue, although at a slower pace, after 8 weeks until about 4 months of age when peak bone mass is attained. Therefore, 9 week old mice used in experiment A can be considered as “young adults”. While it is possible that transient skeletal deficit occurred in the shocked animals, and was corrected by 6 weeks in experiment A, we don't believe this to be the case. In this regard, we have previously reported that while femur BMC nearly doubles between 3 and 4 weeks of age, it only increases 25% between 8 and 13 weeks [51]. Thus, compared to sexually mature 9 week old mice, young growing mice may provide a more sensitive model to investigate the effects of traumatic events due to their rapid growth caused by changes in sex hormones. Therefore, our model will need to be optimized for use in adult mice. Also, since the observed effect manifested early after the stress treatment, and lasted only for a limited period of time, our model may be only valid for acute PTSD. The acute PTSD symptoms are the most common manifestation of PTSD that also holds the most promise for therapy. Still, the far reaching impact of traumatic events in our model on bone development, especially on bone micro-architecture that affects bone quality and bone strength remains to be evaluated.

A correlation analysis for experiment B showed that there were significant negative correlations between the freezing behavior and total body BMC, indicating a connection between increased contextual PTSD-like behavior and decreased bone mass acquisition. Furthermore, we also obtained a significant regression using total body BMC as the dependent variable and an earlier time point freezing behavior as the independent variable, implying that the freezing behavior could be used to a certain degree to predict the amount of bone formation during development. To further understand which individuals might be mainly responsible for the observed correlations and regression, we carried out a stratified analysis of the shocked mice in experiment B. It is known that in humans, not all individuals that are exposed to a trauma develop PTSD. In general, only 30–40% of these individuals will develop PTSD symptoms that persist over a period of time [52], [53]. In animal studies, a similar percentage of mice that have been subjected to electric shocks develop PTSD-like symptoms [40]. Based on this information, we examined the freezing behavior at seven weeks after electric shocks, and divided the shocked mice into three groups: low, medium and high freezing response, in an attempt to assume the high and low freezing response groups having PTSD and non-PTSD, respectively. It was only the high response group, which was 33% of all shocked mice, that had significantly longer freezing time than both the low response and control groups (**Figure 7**), confirming the 30–40% estimate of PTSD prevalence found in previous studies. We also found that the high response group had more BMC reductions in total body, femur and lumbar vertebra than the low response group. These data suggest that the high response group was associated with increased contextual PTSD-like behavior and decreased bone mass acquisition, and both contextual behavioral response and skeletal response were likely part of the PTSD-like symptoms.

In terms of the mechanisms by which traumatic events influences bone formation, it is well established that PTSD leads to activation of Sympathetic Nervous System (SNS) and changes in Hypothalamic-Pituitary-Adrenal (HPA) axis. Molecules such as norepinephrine, neuropeptide-Y, CART and beta-2 adrenergic receptor are the mediators of SNS actions on bone and the messengers of brain-to-bone communication [54], [55], [56]. The PTSD modulation of HPA axis can exert multiple regulatory influences on bone metabolism [57]. PTSD-stimulated response of glucocorticoids such as cortisol through HPA axis has many adverse effects on bone metabolism [14], [58]. PTSD modulation of non-adrenergic activities in HPA axis could lead to a blunted growth hormone (GH) response [59]. GH is an important regulator of IGF-1, which in turn contributes to peak bone mass [51], [60].

In conclusion, electric shocks created long lasting conditioned fear memory in mice. In young mice, electric shocks elicited not only behavioral response but also skeletal response. Prior injection of anxiogenic drug FG-7142 appeared to increase both types of response. Significant negative correlations and regression between behavioral response and skeletal response indicated a strong association between contextual PTSD-like behavior and reductions in bone mass acquisition. Electric shocks coupled with prior injection of anxiognic agents in young mice is thus a good PTSD model, and can be used in studies to determine whether experimental anti-anxiogenic drugs can alleviate the PTSD symptoms as well as mitigate the negative impact of PTSD on bone health.
